# Mapping the Landscape of Anti–Breast Cancer Drug Discovery in Saudi Arabia: A Bibliometric Analysis (2019–2023)

**DOI:** 10.1155/sci5/6991810

**Published:** 2025-12-29

**Authors:** Azizah M. Malebari, Abdulrahman A. Alghelis, Rashad S. Alzahrani, Anfal S. Aljahdali

**Affiliations:** ^1^ Department of Pharmaceutical Chemistry, Faculty of Pharmacy, King Abdulaziz University, Jeddah, 21589, Saudi Arabia, kau.edu.sa; ^2^ Faculty of Pharmacy, King Abdulaziz University, Jeddah, 21589, Saudi Arabia, kau.edu.sa

**Keywords:** anticancer agents, bibliometric analysis, breast cancer, drug discovery, Saudi Arabia, synthetic molecules, VOSviewer

## Abstract

Breast cancer, the most prevalent cancer worldwide, poses a significant public health challenge, especially in the advanced stages. According to the Saudi Health Council (2020), breast cancer is also the leading cancer in Saudi Arabia with recent increases in incidence rates. Over the past two decades, Saudi Arabia has made significant advancements in anti–breast cancer drug discovery driven by increased research funding, improved access to scientific resources, and enhanced education. This study aims to highlight Saudi Arabia’s contributions to this field through a bibliometric analysis of anti–breast cancer drug discovery research published between 2019 and 2023. Using a comprehensive search strategy, 943 publications were retrieved from the Web of Science Core Collection (WoSCC) database and analyzed. Bibliometric tools such as VOSviewer and Microsoft Excel were used to highlight trends in publication output, research areas, and collaboration networks. The results reveal a steady increase in Saudi publications, rising from under 100 in 2019 to over 250 in 2023. The primary research areas were Chemistry (43.69%), Pharmacology/Pharmacy (23.65%), and Biochemistry (22.80%). Elsevier and MDPI were the leading publication platforms, while King Saud University was identified as the primary source of research funding (17.60%). Co‐authorship networks revealed strong collaborations between Saudi institutions and international partners. The most frequent keywords reflected key research priorities within Saudi institutions, “anticancer”, “molecular docking”, and “nanoparticles”. The most commonly studied therapeutic targets were EGFR, Caspase, and VEGF with a diverse range of therapeutic approaches including pyrimidines, nanoparticles, and natural products. This analysis highlights Saudi Arabia’s growing contributions to the field of breast cancer drug discovery and provides a foundation for future research and collaboration.

## 1. Introduction

Breast cancer is the most prevalent form of cancer globally and represents a significant public health concern [1]. It accounts for a significant percentage of cancer‐related deaths, particularly when detected at advanced stages [2, 3]. As reported by the Saudi Health Council in 2020, breast cancer is the most common cancer in Saudi Arabia, with incidence rates continuing to rise [4]. Breast cancer has also surpassed lung cancer as the most frequently diagnosed cancer worldwide, placing considerable burdens on global healthcare systems [5]. Improved early detection, increased public awareness, advanced treatments, and innovative drug development remain essential strategies to reduce mortality, enhance survival rates, and minimize breast cancer’s significant impact on public health [6].

Saudi Arabia has made significant strides in breast cancer drug discovery over the past two decades, primarily due to increased research funding, improved access to scientific resources, high educational standards, and the strategic utilization of abundant natural resources [7]. Recently, Saudi Arabia’s role in drug discovery has expanded further due to advancement in artificial intelligence‐driven research, while continues to revolutionize the field [8]. Research in Saudi Arabia focuses on the development of novel therapeutic agents, including natural products [9–12], semisynthetic derivatives [13–16], and synthetic molecules [17–20]. Although not all discovered compounds progress to clinical trials, these efforts have notably impacted the global drug discovery landscape and have advanced targeted therapies within the country.

Bibliometric analysis provides valuable insights into the landscape of ongoing scientific research, allowing scholars to identify key contributors, emerging trends, and research gaps [21, 22]. Researchers can use these analysis to realign their future research priorities and assess the impact of research outputs by examining patterns of publication, citation, and collaboration. Utilizing bibliometric analysis in medical research also facilitates the dissemination of new knowledge and supports evidence‐based decision‐making [23]. In the context of breast cancer drug discovery, such analysis can highlight underexplored areas of research, foster international collaborations, and inform strategic research directions. However, to date, no bibliometric analysis has focused specifically on breast cancer drug discovery research in Saudi Arabia, highlighting a critical gap in understanding the country’s contributions to this field.

This study aims to conduct a comprehensive bibliometric analysis of Saudi Arabian anti–breast cancer drug discovery research published between 2019 and 2023. By providing an overview of the current research landscape, we intended to identify key trends, leading contributors, and thematic areas within this field. These findings are expected to enhance the visibility of Saudi Arabian research, inspire future studies, foster collaboration, and ultimately improve research outcomes.

## 2. Methods

### 2.1. Data Source and Search Strategy

This study utilized the Web of Science Core Collection (WoSCC) as the primary data source due to its comprehensive coverage and rigorous journal selection criteria, ensuring reliable and high‐quality data for bibliometric analysis. All datasets were collected on October 25, 2023, and focused exclusively on English‐language articles authored in Saudi Arabia. The publication date range of 2019–2023 was selected as representative of recent advancements in anti–breast cancer drug discovery within the country.

A systematic search strategy was employed to ensure selected articles were relevant and covered an appropriate range of scientific fields. The following search expression was designed to comprehensively capture publications related to breast cancer drug discovery:

((TS=(“breast cancer” OR “breast carcinoma” OR breast‐cancer OR breast‐carcinoma) AND TS=(anti‐cancer OR anticancer OR antitumor OR antitumor OR antineoplastic OR anti‐neoplastic OR antitumor OR antitumor OR anti‐proliferative OR antiproliferative OR anti‐proliferation OR antiproliferation OR “anti‐breast cancer” OR “anti breast cancer”)) AND TS=(discovery OR design OR screen OR novel OR new OR synthesis OR extract)).

**Figure 1:**
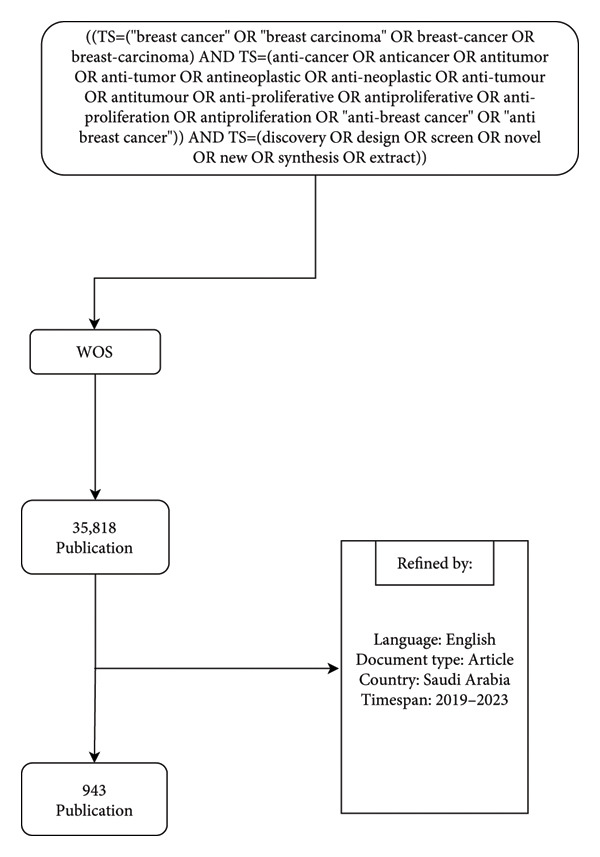
Flowchart of the search strategy and refinement process for anti–breast cancer drug discovery research.

The initial search yielded 35,818 publications, which were subsequently refined to include only English‐language articles authored in Saudi Arabia. This refinement resulted in a final dataset of 943 publications. A flowchart summarizing the search process is presented in Figure 1. Prior to analysis, all records were manually screened for relevance to anti–breast cancer drug discovery.

### 2.2. Data Analysis

Retrieved data were analyzed using VOSviewer (version 1.6.19) for bibliometric network visualization and analysis. Microsoft Excel (2016) was used for data cleaning and summarization.

### 2.3. Bibliometric Network Analysis

VOSviewer was utilized to analyze co‐authorship, keyword co‐occurrence, and citation patterns. In visualized networks, nodes represent authors, keywords, or publications, with node size reflecting metrics such as frequency of occurrence, total link strength, or citation counts. Link thickness indicates relationship intensity (e.g., co‐authorship or co‐citation). Clusters within network represent closely related groups of elements.

### 2.4. Data Cleaning and Visualization

Microsoft Excel was used to clean and organize the retrieved data by removing duplicates and irrelevant records. Charts and tables summarizing publication trends, research focus areas, and collaboration networks were generated for inclusion in the results.

## 3. Results

### 3.1. Global Contributions to Anti–Breast Cancer Drug Discovery

The top 10 countries contributing to anti–breast cancer drug discovery research from 2019 to 2023 are presented in Figure 2(a). China had by far the highest contributions (3280 publications), followed by India (1,875) and the United States (1,663). Saudi Arabia ranked fifth with 943 publications, slightly ahead of Iran (853). Expanding the time frame to 2014–2023 (Figure 2(b)), China remains the top contributor (5643 publications), while Saudi Arabia ranked seventh (1217 publications).

Figure 2Top 10 countries in anti–breast cancer drug discovery publications (a) 2014–2023 and (b) 2019–2023.

**Figure:**
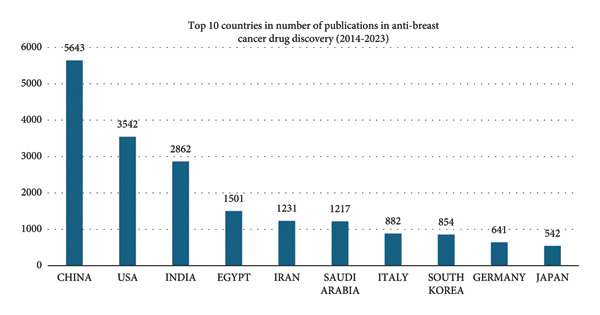
(a)

**Figure:**
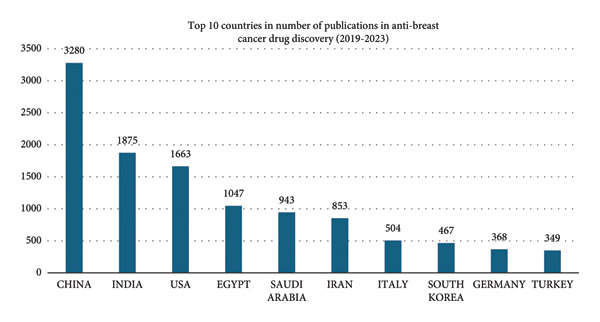
(b)

### 3.2. Growth and Research Areas in Saudi Arabia

**Figure 3:**
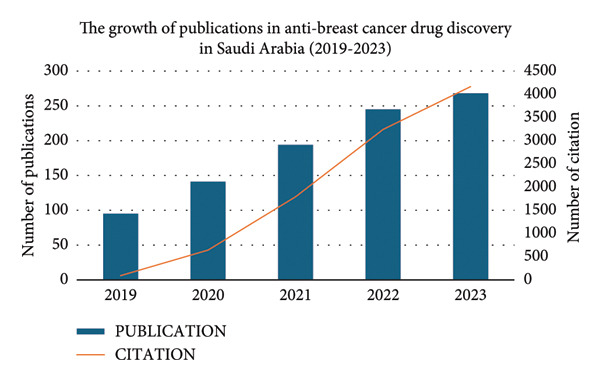
Growth of publications and citations in anti–breast cancer drug discovery in Saudi Arabia (2019–2023).

**Figure 4:**
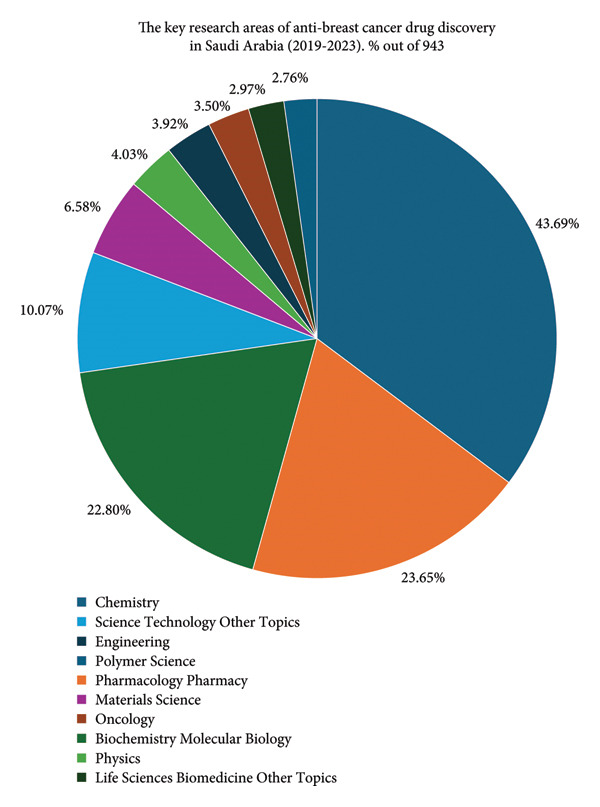
Key research areas in anti–breast cancer drug discovery in Saudi Arabia (2019–2023).

There was a steady annual increase in Saudi Arabian publications and citations between 2019 and 2023 (Figure 3), with publications rising from fewer than 100 in 2019 to over 250 in 2023. Key research areas included Chemistry (43.69%), Pharmacology/Pharmacy (23.65%), and Biochemistry and Molecular Biology (22.80%) (Figure 4). Emerging fields included Materials Science (6.58%) and Oncology (4.03%).

### 3.3. Publishers and Funding Agencies

**Figure 5:**
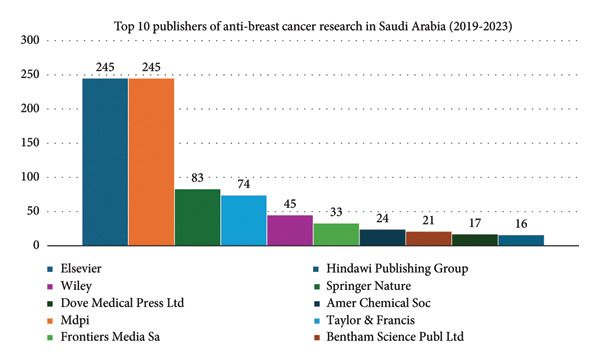
Top 10 publishers of anti–breast cancer research in Saudi Arabia (2019–2023).

**Figure 6:**
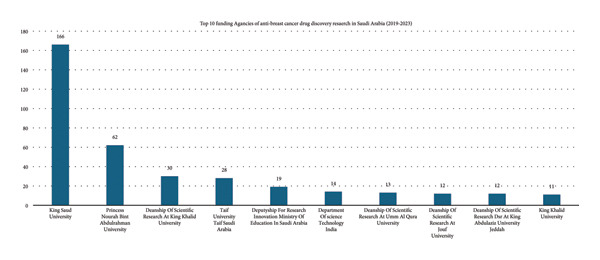
Top 10 funding agencies for anti–breast cancer drug discovery research in Saudi Arabia (2019–2023).

Between 2019 and 2023, the leading publishers were Elsevier and MDPI, each with 245 publications (Figure 5). Other notable publishers included Wiley (83 publications), Dove Medical Press Ltd (74 publications), and Springer Nature (45 publications). Key funding sources were King Saud University (17.60%), Princess Nourah Bint Abdulrahman University (6.58%), and King Khalid University (3.18%) (Figure 6). Additional support came from Taif University (2.97%) and the Deputyship for Research Innovation at the Ministry of Education (2.02%).

### 3.4. Co‐Authorship Networks

Figure 7(a) highlights co‐authorship networks among institutions, with King Saud University and King Abdulaziz University occupying central nodes. Collaboration networks extended internationally, involving institutions such as Kuwait University and Jamia Hamdard. Author‐specific collaborations highlighted several prominent researchers, including M. Y. Alfaifi, W. M. Eldehna, and N. Abutaha (Figure 7(b)).

Figure 7Co‐authorship network between (a) organizations and (b) authors in anti–breast cancer research in Saudi Arabia.

**Figure:**
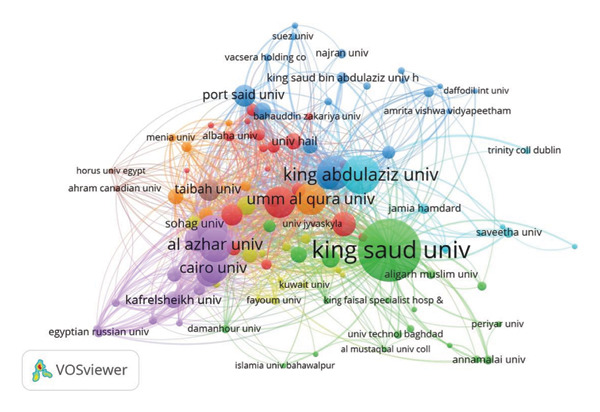
(a)

**Figure:**
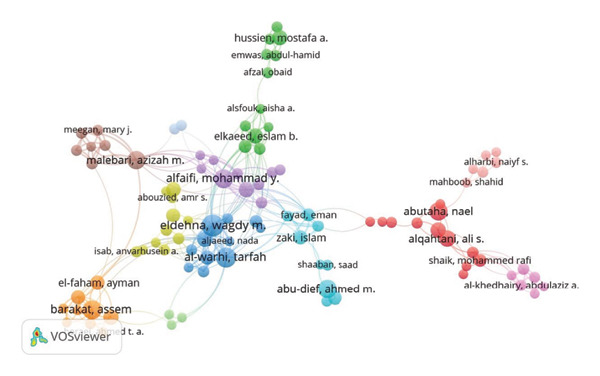
(b)

### 3.5. Co‐Citation Networks

The author co‐citation network shows that Mosmann, T. and Abdel‐Rahman, L. H. were the most frequently co‐cited authors, with additional clusters formed around Supuran, C. T., Gomha, S. M., and Eldehna, W. M (Figure 8(a)). Similarly, the reference co‐citation network identified Mosmann, T. (1983) and Abdel‐Rahman, L. H. (2016) as the most frequently cited studies (Figure 8(b)), alongside work by Bayazeed, A. A. (2020) and Supuran, C. T. (2018).

Figure 8Co‐citation network of (a) authors and (b) references in anti–breast cancer research in Saudi Arabia.

**Figure:**
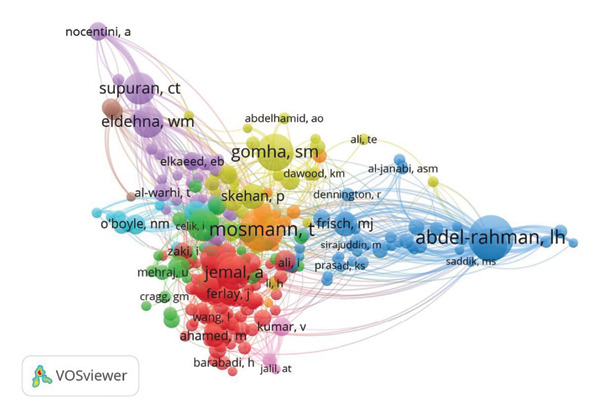
(a)

**Figure:**
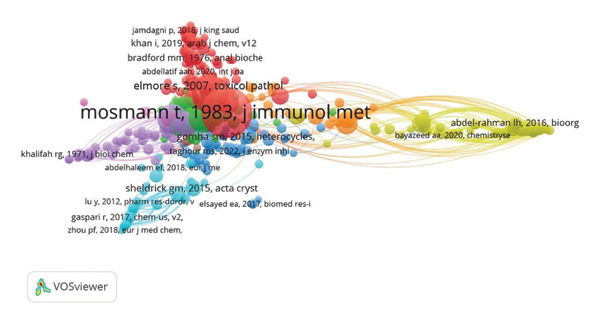
(b)

### 3.6. Keyword Co‐Occurrence

Frequently occurring keywords included anticancer, molecular docking, apoptosis, and nanoparticles (Figure 9(a)), reflecting prevalent themes in drug discovery studies. Other common terms included green synthesis, gold nanoparticles, and biosynthesis.

Figure 9Co‐occurrence network of (a) keywords and (b) authors’ keywords in anti–breast cancer drug discovery research.

**Figure:**
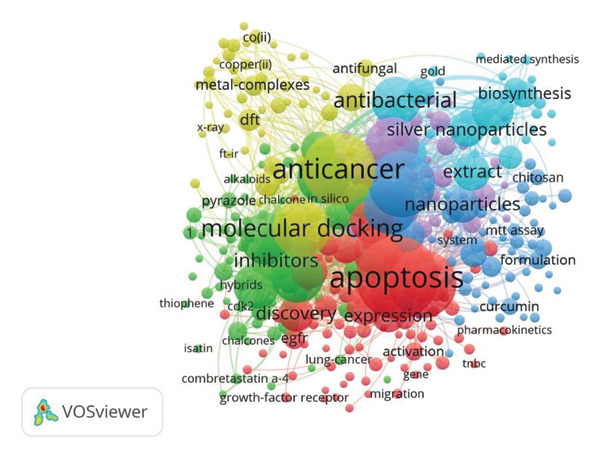
(a)

**Figure:**
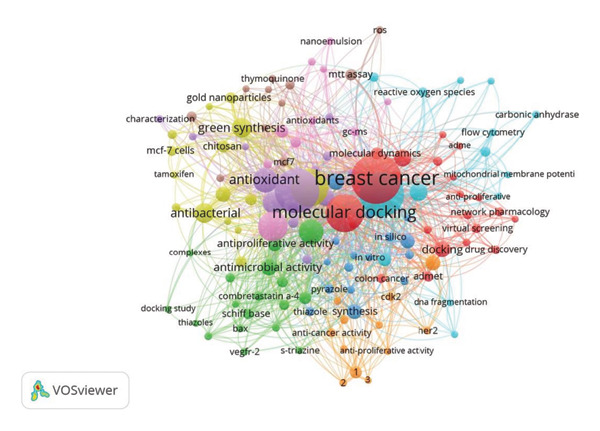
(b)

The most frequent referenced experimental methodologies included terms such as MCF‐7 cells, MTT assay, and flow cytometry (Figure 9(b)); these terms are commonly associated with cytotoxicity testing and cellular mechanism studies. Additional frequent keywords included reactive oxygen species (ROS) and gene expression.

### 3.7. Leading Saudi Affiliations, Top Journals, and Most Cited Articles

Analysis of institutional contributions revealed that King Saud University had the greatest contribution to breast cancer drug discovery research in Saudi Arabia with 302 publications (32.03%). It was followed by King Abdulaziz University (132; 13.99%) and King Khalid University (108; 11.03%). Other significant contributors included Umm Al‐Qura University (98; 10.39%), Taif University (91; 9.65%), Princess Nourah Bint Abdulrahman University (88; 9.33%), and Prince Sattam Bin Abdulaziz University (61; 6.47%) (Table 1).

**Table 1:** Top 10 Saudi affiliations contributing to anticancer drug discovery research.

Saudi affiliations	No. of affiliated papers	% of 943
KING SAUD UNIVERSITY	302	32.025
KING ABDULAZIZ UNIVERSITY	132	13.998
KING KHALID UNIVERSITY	108	11.029
UMM AL QURA UNIVERSITY	98	10.392
TAIF UNIVERSITY	91	9.650
PRINCESS NOURAH BINT ABDULRAHMAN UNIVERSITY	88	9.332
PRINCE SATTAM BIN ABDULAZIZ UNIVERSITY	61	6.469
QASSIM UNIVERSITY	55	5.832
TAIBAH UNIVERSITY	48	5.090
AL JOUF UNIVERSITY	47	4.984

The most popular journal was Molecules with 77 publications (IF: 4.6), followed by Journal of Molecular Structure with 31 publications (IF: 3.8) and Arabian Journal of Chemistry with 27 publications (IF: 6.0). Prominent local journals included Journal of King Saud University Science (18 publications, IF: 3.8) and the Saudi Journal of Biological Sciences (15 publications, IF: 4.4) (Table 2).

**Table 2:** The top journals in anti–breast cancer drug discovery research in Saudi Arabia.

Journal	Publisher	No. of published papers	If
MOLECULES	MDPI	77	4.6
JOURNAL OF MOLECULAR STRUCTURE	Elsevier	31	3.8
ARABIAN JOURNAL OF CHEMISTRY	Elsevier	27	6.0
PHARMACEUTICALS	MDPI	24	4.6
ACS OMEGA	American Chemical Society	22	4.1
BIOORGANIC CHEMISTRY	Elsevier	21	5.1
JOURNAL OF BIOMOLECULAR STRUCTURE DYNAMICS	Taylor & Francis	20	4.4
JOURNAL OF KING SAUD UNIVERSITY SCIENCE	Elsevier	18	3.8
JOURNAL OF ENZYME INHIBITION AND MEDICINAL CHEMISTRY	Taylor & Francis	16	5.6
SCIENTIFIC REPORTS	Nature Publishing Group	16	4.6
CHEMISTRYSELECT	Wiley‐VCH	15	2.1
SAUDI JOURNAL OF BIOLOGICAL SCIENCES	Elsevier	15	4.4
PHARMACEUTICS	MDPI	14	5.4
SAUDI PHARMACEUTICAL JOURNAL	Elsevier	12	4.1
INTERNATIONAL JOURNAL OF MOLECULAR SCIENCES	MDPI	11	5.6
POLYMERS	MDPI	11	5.0
APPLIED SCIENCES BASEL	MDPI	10	2.8
JOURNAL OF DRUG DELIVERY SCIENCE AND TECHNOLOGY	Elsevier	10	5.0
JOURNAL OF MOLECULAR LIQUIDS	Elsevier	10	6.0
FRONTIERS IN CHEMISTRY	Frontiers Media	9	5.5

The most cited article was “Hesperidin Loaded on Gold Nanoparticles as a drug delivery system for a successful biocompatible, anti‐cancer, anti‐inflammatory and phagocytosis inducer mode” by Sulaiman et al. (2020), with 129 citations. This was followed by a study on palladium nanoparticles by Sonbol et al. (2021) (106 citations) and research on gold nanoparticles synthesized using *Fusarium solani* conducted by Clarance et al. (2020) (98 citations). Additional notable contributions involved research on ZnO/Reduced Graphene Oxide Nanocomposites (Ahamed et al., 2021; 86 citations), metal–organic frameworks (El‐Bindary et al. 2020; 86 citations), and Schiff base complexes (Abu‐Dief et al. 2021; 77 citations) (Table 3).

**Table 3:** The top 10 most cited articles in anti–breast cancer drug discovery from Saudi Arabia.

Article title	Author	Times cited, WoS core	Publisher	Journal	Publication year
Hesperidin Loaded on Gold Nanoparticles as a Drug Delivery System for a Successful Biocompatible, Anti‐Cancer, Anti‐Inflammatory and Phagocytosis Inducer Model	Sulaiman et al.	129	NATURE PORTFOLIO	Scientific Reports	2020
*Padina boryana* mediated green synthesis of crystalline palladium nanoparticles as potential nanodrug against multidrug resistant bacteria and cancer cells	Sonbol et al.	106	NATURE PORTFOLIO	Scientific Reports	2021
Green synthesis and characterization of gold nanoparticles using endophytic fungi Fusarium solani and its in vitro anticancer and biomedical applications	Clarance et al.	98	ELSEVIER	Saudi Journal of Biological Sciences	2020
SnO2‐Doped ZnO/Reduced Graphene Oxide Nanocomposites: Synthesis, Characterization, and Improved Anticancer Activity via Oxidative Stress Pathway	Ahamed et al.	86	DOVE MEDICAL PRESS LTD	International Journal of Nanomedicine	2021
Metal‐organic frameworks as efficient materials for drug delivery: Synthesis, characterization, antioxidant, anticancer, antibacterial and molecular docking investigation	El‐Bindary et al.	86	WILEY	Applied Organometallic Chemistry	2020
Green‐Synthesized Silver Nanoparticles Induced Apoptotic Cell Death in MCF‐7 Breast Cancer Cells by Generating Reactive Oxygen Species and Activating Caspase 3 and 9 Enzyme Activities	Ullah et al.	84	HINDAWI LTD	Oxidative Medicine and Cellular Longevity	2020
Synthesis and intensive characterization for novel Zn(II), Pd(II), Cr(III) and VO(II)‐Schiff base complexes; DNA‐interaction, DFT, drug‐likeness and molecular docking studies	Abu‐Dief et al.	77	ELSEVIER	Journal of Molecular Structure	2021
Enhancement of the tail hydrophobic interactions within the carbonic anhydrase IX active site via structural extension: Design and synthesis of novel N‐substituted isatins‐SLC‐0111 hybrids as carbonic anhydrase inhibitors and antitumor agents	Eldehna et al.	77	ELSEVIER	European Journal of Medicinal Chemistry	2019
Catalytic, antioxidant and anticancer activities of gold nanoparticles synthesized by kaempferol glucoside from Lotus leguminosae	Oueslati et al.	72	ELSEVIER	Arabian Journal of Chemistry	2020
Synthesis, structures, DNA‐binding and anticancer activities of some copper(I)‐phosphine complexes	Mashat et al.	71	PERGAMON‐ELSEVIER SCIENCE LTD	Polyhedron	2019

### 3.8. Popular Targets in Drug Discovery

The most studied molecular targets in breast cancer drug discovery included epidermal growth factor receptor (EGFR), vascular endothelial growth factor receptor‐2 (VEGFR‐2), cyclin‐dependent kinases (CDKs), tubulin, and hormone receptors (ERα and ERβ) (Table 4). EGFR inhibitors are the most frequently studied compounds, often featuring pyrimidine‐2‐one, pyrazole, benzimidazole, triazole, and thiophene sulfonamides [24, 25, 28, 29, 35]. Several compounds, such as thiazolidin‐4‐ones and ureido‐benzothiophenes, exhibit dual inhibition of EGFR/CDK2 and EGFR/VEGFR2, respectively [42, 66]. Tubulin inhibitors included β‐lactams (2‐azetidinones), coumarins, and acrylic acid esters, while CDK inhibitors included quinazolinones, oxindole‐indole conjugates, and benzofuran hybrids [47, 48, 52–54, 56, 59]. Some CDK2‐targeting compounds were also found to inhibit GSK‐3β [60]. Hormone receptor inhibitors primarily targeted ERα and Erβ, including curcumin, vanillin‐based indolin‐2‐one, and coumarin‐chalcone derivatives [62, 63, 65]. Multitarget inhibitors, such as 2‐mercaptobenzoxazole derivatives, exhibited activity against EGFR, HER2, VEGFR2, and CDK2 offering enhanced therapeutic efficacy and potential for overcoming resistance mechanisms [55].

**Table 4:** Key molecular targets and associated pharmacophores in breast cancer drug discovery research.

Biologically active novel derivatives	Protein targets	Source
pyrimidine‐2‐one(thionic) or pyrazole	EGFR	[24]
Benzimidazole, 1,2,4‐triazole, and s‐triazine derivatives	EGFR	[25]
3′‐(4‐(Benzyloxy)phenyl)‐1′‐phenyl‐5‐(heteroaryl/aryl)‐3,4‐dihydro‐1′H,2H‐[3,4′‐bipyrazole]‐2‐carboxamides	EGFR	[26]
(T‐1‐MMPA) theobromine (meta methoxy phenyl)acetamide derivative)	EGFR	[27]
1*H*‐pyrazole‐1‐carbothioamide derivatives	EGFR	[28]
4‐(2‐arylidenehydrazineyl)thienopyrimidine derivatives	EGFR	[29]
Isoxazolidine derivatives	EGFR	[30]
Ethylidenehydrazineylthiazol‐4(5H)‐ones	EGFR	[17]
Thiophene Sulfonamide Derivatives	EGFR	[31]
hydrazone‐isatin derivatives	EGFR	[32]
3,5‐bis(substituted benzylidene)‐1‐ethylpiperidin‐4‐one analogs	EGFR	[33]
benzofuran‐pyrazole‐thiazolidinone	EGFR	[34]
Pyrazole‐thiazo‐4‐one	EGFR	[35]
1,3‐thiazolidin‐4‐ones	EGFR‐CDK2	[36]
bis‐(6‐pyrazolyltriazolo‐thiadiazine) derivatives	EGFR/CDK‐2	[37]
chalcones incorporating thiadiazolyl isoquinoline	EGFR/CDK‐2	[38]
Indole‐2‐Carboxamides	EGFR and CDK2	[39]
Alkylsulfanylpyridazino[4,5‐b]indole compounds	EGFR and its downstream PI3K–AKT	[40]
Thiazolyl‐Pyrazoline Derivatives	EGFR/HER2	[41]
ureido benzothiophenes	EGFR/VEGFR2	[42]
5,5‐diphenylimidazolidine‐2,4‐dione derivatives	EGFR/VEGFR2	[43]
2‐substituted‐quinoxaline analogs	topoisomerase II and EGFR proteins.	[44]
5‐cyano‐6‐oxo‐pyridine‐based sulfonamides	EGFR) and carbonic anhydrase (CA)	[45]
Enamide fluorinated‐Schiff base derivatives	Tubulin	[46]
vinyl‐beta‐lactams (2‐azetidinones)	Tubulin	[47]
beta‐lactams (2‐azetidinones)	Tubulin	[48]
*cis* restricted 1,2,4‐triazole analogs of combretastatin A‐4	Tubulin	[49]
Vinyl amide‐, imidazolone‐, and triazinone‐linked combretastatin A‐4 analogs	Tubulin	[50]
coumarin derivatives	Tubulin	[51]
Acrylic acid and acrylate ester derivatives	Tubulin	[52]
3‐fluoro and 3,3‐difluoro substituted beta‐lactams	Tubulin	[53]
3‐chloro‐beta‐lactams and 3,3‐dichloro‐beta‐lactams (2‐azetidinones	Tubulin	[54]
2‐Mercaptobenzoxazole	EGFR, HER2, VEGFR2, and CDK2	[55]
Quinazolin‐4(3H)‐one derivatives	CDK2, HER2, EGFR	[56]
2‐Mercaptobenzoxazole derivatives	EGFR, HER2, VEGFR2, and CDK2	[55]
4,6,7,8‐tetrahydroquinolin‐5(1H)‐ones	EGFR, HER‐2, PDGFR‐β, and VEGFR‐2	[57]
3‐Hydrazonoindolin‐2‐One	CDK2	[58]
Oxindole–Indole Conjugates	CDK4	[59]
oxindole/benzofuran hybrids	CDK2 and GSK‐3β	[60]
curcumin derivatives	Erα receptors	[61]
Vanillin‐Based Indolin‐2‐one Derivative	Erα receptors	[62]
coumarin‐chalcone derivatives	ERα and ERβ receptors	[63]
Dihydropyrimidine‐pregnenolone analogs	ERα and ERβ receptors	[64]
Pyrimidine‐2‐sulfonamide derivatives based on the 2*H*‐chromen‐2‐one	ERα and 4CDK2/Cyclin	[65]

### 3.9. Gene Targets and Biomarker‐Based Approaches

The retrieved literature from Saudi Arabia revealed a growing emphasis on breast cancer biomarker discovery, with frequent focus on gene expression alterations, signaling regulators, and apoptotic mediators. Among the most recurrently studied genes were TP53, BRCA1/2, and PIK3CA, followed by emerging markers such as SLC31A1 and GLO1, highlighting their roles in apoptosis, DNA repair, and chemoresistance [67–70]. Several studies demonstrated biomarker modulation via natural products (e.g., *Avicennia marina*, *Euphorbia abyssinica*), green‐synthesized nanoparticles (e.g., CuONPs, Pd‐NPs), and synthetic derivatives, which collectively induced mitochondrial apoptosis, inhibited PI3K/AKT or Wnt/β‐catenin signaling, and restored TP53 function [67, 71, 72].

## 4. Discussion

### 4.1. Overview of Saudi Arabia’s Contributions to Anti–Breast Cancer Research

This bibliometric analysis provides a comprehensive overview of Saudi Arabia’s contributions to anti–breast cancer drug discovery research. The results highlight Saudi Arabia’s active engagement, positioning it as one of the top global contributors in this field, with steady increase in publications and citations over the past 5 years. These findings reflect the growing emphasis on cancer research in the Kingdom, driven by increased funding, international collaboration, and multidisciplinary research efforts. The rise in publication numbers is likely associated with increasing government investment in cancer research.

The growth in publications related to chemistry, pharmacology/pharmacy, and biochemistry highlights the multidisciplinary nature of anticancer research in Saudi Arabia [67, 73, 74]. Such disciplinary diversity is essential for addressing the complexities of breast cancer, advancing the development of novel therapeutic agents and drug delivery systems. Emerging fields, such as materials science and nanotechnology, further emphasize the Kingdom’s commitment to modern research methodologies and international collaboration [75–77].

Funding, collaboration, and institutional contributions are key drivers of Saudi Arabia’s productivity in the context of anti–breast cancer drug discovery research. In particular, King Saud University and Princess Nourah Bint Abdulrahman University play pivotal roles in fostering international research partnerships, as reflected in their central positions within co‐authorship networks [67, 75, 78–81]. Leading Saudi universities, including King Abdulaziz, King Khalid, and Umm Al‐Qura, have also contributed significantly, highlighting the strength of the country’s research infrastructure [82–84]. Elsevier and MDPI were identified as preferred publishing platforms, with additional publications in Wiley, Springer Nature, Dove Medical Press, and Hindawi emphasizing the multidisciplinary nature of cancer research [73, 75, 76, 85–87]. Strong institutional and governmental support, particularly from King Saud University and the Ministry of Education’s Deputyship for Research Innovation, further reinforce Saudi Arabia’s growing role in global cancer research and drug discovery [87–89].

### 4.2. Key Institutions, Authors, and Publishing Platforms

The co‐authorship network also highlights the leading role of Saudi Arabian institutions in international collaboration: King Saud University and King Abdulaziz University demonstrated strong collaborative ties both domestically and internationally, fostering partnerships with regional entities like Cairo University and Al‐Azhar University, as well as global counterparts such as Jamia Hamdard and Trinity College Dublin [40, 47, 48, 60, 67, 71, 75, 78, 90–98]. King Faisal Specialist Hospital & Research Center also plays a crucial role in translational research, bridging academic discoveries and clinical applications [99–101]. At the individual level, researchers such as M. Y. Alfaifi, W. M. Eldehna, and N. Abutaha hold prominent positions within the co‐authorship network, reflecting their significant contributions and frequent collaborations [102–109].

The author co‐citation network highlights key contributors and foundational references that have shaped breast cancer drug discovery research in Saudi Arabia. Thomas Mosmann stands out as a central figure due to his seminal work on the MTT assay, a widely used technique for assessing cell viability in cancer studies [110]. Similarly, the contributions of Abdel‐Rahman, L. H. to bioorganic chemistry and the synthesis of anticancer agents have strengthened the connection between synthetic chemistry and cancer research [111–113]. Other notable authors, such as Supuran, C. T. and Eldehna, W. M., form distinct clusters within the co‐citation network, reflecting specialized subfields in breast cancer drug discovery [105, 106, 114, 115].

The reference co‐citation network further underscores the influence of Mosmann, T. His 1983 study remains a cornerstone of cancer research due to its lasting impact on drug screening methodologies [110]. The presence of highly cited interdisciplinary works highlights the importance of integrating immunology, chemistry, and oncology to advance therapeutic strategies [40, 116, 117]. These networks illustrate the collaborative and global nature of breast cancer research and emphasize the collective effort required to develop innovative cancer treatments.

### 4.3. Research Themes and Methodologies in Breast Cancer Drug Discovery

Keyword co‐occurrence analysis identified dominant research themes in anti–breast cancer drug discovery in Saudi Arabia, highlighting the strong focus on nanotechnology, molecular docking, and apoptosis‐related studies [75, 85, 118]. The frequent occurrence of terms such as gold nanoparticles, green synthesis, and biosynthesis underscores the emphasis on eco‐friendly and nanomaterial‐based drug development strategies [67, 119, 120]. Methodological keywords such as MCF‐7 cells, MTT assay, and flow cytometry indicate a reliance on well‐established in vitro models and cytotoxicity testing techniques. Additionally, the presence of terms related to oxidative stress and gene expression suggests growing interest in understanding drug‐induced cellular responses [12, 121–123]. This provides further support for multidisciplinary approaches to cancer research.

The most highly cited Saudi Arabian drug discovery publications have primarily focused on nanotechnology, green chemistry, and targeted therapies. Among them, Sulaiman et al. (2020) hold the highest citation count (129) for their study on gold nanoparticles loaded with the flavonoid glycoside hesperidin. Their research demonstrated enhanced cytotoxicity against breast cancer cells with no significant in vivo toxicity, making it a promising candidate for cancer therapy [78]. Following closely, Sonbol et al. (2021) (106 citations) synthesized palladium nanoparticles using *Padina boryana* extract, reporting significant antibacterial, antibiofilm, and anticancer activities against MCF‐7 breast cancer cells, highlighting their multifunctional therapeutic potential [67]. Clarance et al. (2020) (98 citations) utilized *Fusarium solani*–mediated gold nanoparticles, which effectively induced apoptosis in MCF‐7 and cervical (HeLa) cancer cells by arresting them in the sub‐G0 and G1 phases of the cell cycle [51]. Ahamed et al. (2021) (86 citations) explored anticancer potential of SnO_2_‐doped ZnO/reduced graphene oxide nanocomposites, demonstrating their superior selectivity in targeting MCF‐7 cells while sparing normal breast epithelial (MCF10A) cells [76]. Similarly, El‐Bindary et al. (2020) (86 citations) developed a metal–organic framework (ZIF‐8) encapsulating doxorubicin, leading to pH‐sensitive drug release and enhanced cytotoxicity against both MCF‐7 and HepG‐2 cancer cells, in addition to significant antibacterial properties [85].

Ullah et al. (2020) (84 citations) utilized *Fagonia indica* extracts for the green synthesis of silver nanoparticles, which effectively induced apoptosis in MCF‐7 cells through ROS generation and caspase activation, highlighting their potential as nanomedicine candidates [87]. Abu‐Dief et al. (2021) (77 citations) synthesized Schiff base metal complexes that exhibited strong DNA‐binding affinities and targeted protein kinase inhibition, with Pd(II) complexes demonstrating potent cytotoxic activity against breast cancer cells [124].

Eldehna et al. (2019) (77 citations), designed N‐substituted isatin‐SLC‐0111 hybrids that selectively inhibit carbonic anhydrase IX inducing apoptosis, cell cycle arrest, and VEGFR‐2 inhibition in MDA‐MB‐231 and MCF‐7 cells [105]. Oueslati et al. (2020) (72 citations) synthesized gold nanoparticles from *Lotus leguminosae*, which exhibited mild cytotoxicity against MCF‐7 cells and demonstrating catalytic activity in reducing *p*‐nitrophenol [125]. Finally, Mashat et al. (2019) (71 citations), developed copper(I) complexes featuring phenanthroline‐phosphine ligands, which exhibited strong DNA‐binding affinities, lipophilicity variations, and potent anticancer activity in breast and prostate cancer cell lines [97].

### 4.4. Advances in Molecular Targeting and Pharmacophore Design

Breast cancer is a molecularly heterogeneous disease, with treatment strategies guided by specific molecular targets such as receptor tyrosine kinases (e.g., EGFR and HER2), hormonal receptors (ER and PR), and key regulatory proteins involved in tumor progression, including PIK3CA, CDKs, and tubulin [5, 26, 27, 55, 60]. Advances in drug discovery have led to the development of novel pharmacophores designed to selectively inhibit these targets, enhancing treatment efficacy and reducing resistance [26, 27, 55]. EGFR is the most extensively studied target, with pyrimidine‐2‐one(thionic), pyrazole, benzimidazole, and triazole derivatives emerging as promising inhibitors [24, 25, 28, 29, 35]. Notably, benzofuran‐pyrazole‐thiazolidinone, hydrazone, and isoxazolidine compounds have shown strong EGFR inhibition, effectively blocking downstream signaling cascades involved in tumor growth [30, 32, 34]. The development of dual‐target inhibitors, such as ureido‐benzothiophenes and 5,5‐diphenylimidazolidine‐2,4‐dione derivatives, further underscore the growing interest in multitargeted therapies that simultaneously inhibit EGFR and VEGFR‐2, offering enhanced antiproliferative and antiangiogenic effects [42, 43].

VEGFR‐2 inhibitions have also been a major focus in breast cancer drug discovery due to its pivotal role in tumor angiogenesis [57, 126]. Several pharmacophores including thiophene sulfonamides, ethylidene hydrazine yl thiazolones, and 4‐(2‐arylidene hydrazine yl)thienopyrimidines have exhibited promising VEGFR‐2 inhibitory activity, disrupting tumor vascularization and impairing cancer cell proliferation [17, 31]. Notably, 4,6,7,8‐tetrahydroquinolin‐5(1H)‐ones have demonstrated the ability to target multiple receptors, including EGFR, HER‐2, PDGFR‐β, and VEGFR‐2, reinforcing the trend toward broad‐spectrum inhibitors that enhance therapeutic efficacy and address resistance mechanisms [57].

In addition to receptor tyrosine kinases, CDKs, serine/threonine kinase, have emerged as crucial targets in breast cancer therapy due to their role in regulating cell cycle progression through cyclin activation [58]. Dysregulation or overexpression of CDKs and cyclins is associated with various cancers, making them key targets for cancer therapy [39]. Pharmacophores such as 1,3‐thiazolidin‐4‐ones and bis‐(6‐pyrazolyltriazolo‐thiadiazine) derivatives can effectively inhibit CDK2, providing support for strategies that target dysregulated cell cycle pathways in tumor cells [36–38]. Similarly, oxindole‐indole conjugates have been reported to selectively inhibit CDK4, demonstrating their potential for cell cycle arrest in cancer therapy [59]. The presence of dual inhibitors such as alkyl sulfanyl pyridazino[4,5‐b]indole compounds, which target both the EGFR and the PI3K–AKT pathway, further supports the use of multitarget strategies in kinase inhibition [40].

Tubulin, an essential component of the cytoskeleton, is another crucial target in anticancer therapy due to its essential role in maintaining cytoskeletal integrity and facilitating cell division [46]. Several β‐lactam derivatives, including 3‐fluoro and 3,3‐difluoro‐substituted β‐lactams, 3‐chloro and 3,3‐dichloro‐β‐lactams, and vinyl‐β‐lactams (2‐azetidinones), have been shown to inhibit tubulin, disrupting microtubule polymerization and leading to mitotic arrest in cancer cells [47, 48, 53, 54]. Combretastatin A‐4 analogs, such as *cis*‐restricted 1,2,4‐triazole derivatives and imidazolone, provide further support for the effectiveness of targeting tubulin to impair cancer cell division [49, 50].

HER2‐targeted therapies have also gained in prominence, with pharmacophores such as quinazolin‐4(3H)‐one derivatives and thiazolyl‐pyrazoline compounds demonstrating significant HER2 inhibition [41, 56]. The development of dual inhibitors such as 2‐mercaptobenzoxazole derivatives, which target EGFR, HER2, VEGFR2, and CDK2, exemplifies the growing trend toward multitargeted cancer therapies designed to overcome a range of resistance mechanisms [55]. Targeting estrogen receptors (ERα and ERβ) continues to be a key approach in treating hormone‐dependent breast cancer. Several novel pharmacophores have been developed to inhibit ER activity, including curcumin derivatives, vanillin‐based indolin‐2‐one compounds, and dihydropyrimidine‐pregnenolone analogs, which selectively modulate ERα [61, 62, 64]. Additionally, coumarin‐chalcone hybrids have also been shown to inhibit both ERα and ERβ, thus offering broader therapeutic potential [63]. Pyrimidine‐2‐sulfonamide derivatives that incorporate a 2H‐chromen‐2‐one scaffold have been used to expand the range of ER‐targeting agents while also inhibiting CDK2/Cyclin [65]. These findings collectively highlight the integral role of pharmacophore design in targeting diverse molecular pathways in cancer progression. The relationship between the structural features of pharmacophores and their molecular targets continues to drive advancements in cancer drug discovery, particularly with regard to tailoring cancer therapies to address specific mechanisms of the disease.

### 4.5. Integration of Biomarkers in Saudi Drug Discovery Efforts

In recent years, Saudi Arabia’s contributions to biomarker‐based breast cancer research have grown substantially, particularly in the context of gene expression profiling and molecular target validation. Biomarkers including tumor suppressor genes, DNA repair regulators, apoptosis mediators, and signaling proteins have enabled precision oncology by linking molecular alterations to therapeutic vulnerability [6]. This expanding body of Saudi research spans classical markers such as TP53, BRCA1/2, and PIK3CA, alongside emerging targets related to cuproptosis, inflammation, and multidrug resistance, collectively underscoring the growing integration of biomarker discovery into the national cancer drug development pipeline [9, 87]. More recently, SLC31A1, a cuproptosis‐related gene, has emerged as a novel breast cancer biomarker with diagnostic and prognostic significance, showing strong upregulation driven by hypomethylation and association with poor survival and immune cell infiltration [69]. Additionally, Canagliflozin has been shown to upregulate BRCA1 while suppressing mTOR‐mediated inflammatory and pyroptotic signaling, reinforcing its therapeutic potential in breast cancer management [68].

In addition to gene regulators, proliferative markers such as Ki67 and oncogenic drivers like HER2 have also been highlighted in nanotherapeutic evaluations of MoS_2_/VS_2_ nanocomposites, demonstrating promising cytotoxicity against breast cancer cells [127]. Beyond classical biomarkers, PARP1 has been the focus of structure‐ and ligand‐based screening in Saudi studies targeting BRCA‐mutated tumors, resulting in the discovery of novel heterocyclic inhibitors distinct from approved PARP inhibitors scaffolds [128]. Expanding into triple‐negative subtypes, Human CK2*α* kinase has also been proposed as a viable target, with Scutellarein derivatives exhibiting strong binding affinity and pharmacokinetic potential [129].

Several Saudi studies have highlighted TP53 as a central tumor suppressor in breast cancer, particularly for its role in regulating apoptosis, cell cycle arrest, and drug sensitivity. Phytochemicals from *Avicennia marina*, including stigmasterol and betulinic acid, showed strong binding to TP53, AKT1, CTNNB1, IL6, and TNF in network pharmacology analyses, notably restoring TP53‐mediated apoptosis and suppressing Wnt/β‐catenin‐driven EMT [130]. Similarly, *Padina boryana*–derived palladium nanoparticles (Pd‐NPs) triggered apoptosis in MCF‐7 cells via mitochondrial pathways, with upregulation of p53, Bax, caspase‐3, and caspase‐9, showing promising potential for overcoming multidrug resistance [67]. Copper‐based nanoparticles derived from *Eucalyptus globulus* and quercetin‐loaded CuO‐ChNPs have both demonstrated p53‐mediated apoptosis in breast cancer cells, driven by ROS generation, mitochondrial depolarization, cytochrome c release, and caspase activation, alongside reduced PCNA expression and tumor proliferation in vivo [71, 72]. While natural agents target p53 via oxidative and mitochondrial stress, synthetic scaffolds have also effectively modulated p53‐dependent apoptosis. A novel benzofuran–pyrazol thiazolidinone hybrid exhibited strong EGFR inhibition and G1/S arrest in HeLa cells, with p53‐dependent upregulation of Bax/Bcl‐2 and caspases‐3/7, suggesting its potential for application in breast cancer therapy [34].

Saudi studies have identified PIK3CA as a key biomarker in breast cancer progression and resistance. In chemically characterized models, α‐aminophosphonates and arylidene derivatives suppressed PIK3CA, Bcl‐2, and PCNA, while upregulating BAX and PIK3R1, indicating PI3K/AKT inhibition and mitochondrial apoptosis [131]. Extending the resistance axis, proteomic studies identify glyoxalase 1 (Glo1) overexpression as a key contributor to multidrug resistance in breast cancer by detoxifying methylglyoxal and suppressing apoptosis. Its elevated levels correlate with reduced drug efficacy and survival, suggesting that Glo1 inhibition may restore apoptotic signaling and improve treatment response [70]. In parallel, natural product–based approaches using *Euphorbia abyssinica*–derived triterpenes have shown cytotoxicity in MCF‐7 cells by targeting regulators such as EGFR, PIK3CA, and PTGS2, thereby disrupting PI3K/AKT, Wnt, and VEGF pathways. These findings underscore the therapeutic potential of biomarker‐guided, multitarget strategies to overcome resistance and inhibit breast cancer progression [132]. In line with these experimental approaches, in silico drug repurposing strategies in Saudi research have pinpointed TP53, BRCA1/2, PIK3CA, and PARP1 as high‐priority targets for re‐sensitizing breast tumors to conventional therapies. By integrating transcriptomic and proteomic profiles with docking predictions, these approaches forecast potential repositioned agents for DNA damage response (DDR) modulation and apoptotic reactivation [117].

### 4.6. Summary and Future Directions

This bibliometric and scientific mapping analysis confirms that Saudi Arabia has emerged as a significant contributor to anti–breast cancer drug discovery, with increasing research output, international collaboration, and institutional investment. Alongside synthetic pharmacophore development and nanotechnology, recent Saudi studies have demonstrated a strong shift toward biomarker‐guided strategies, particularly targeting regulators such as TP53, BRCA1/2, PIK3CA, HER2, and PARP1.

Future directions should emphasize the integration of patient‐derived models, multi‐omics platforms, and clinical validation of biomarker–drug relationships to improve the precision and efficacy of breast cancer therapeutics. Strengthening national databases, expanding collaborative networks, and investing in next‐generation technologies such as AI‐driven drug repurposing and nanomedicine delivery systems will be critical for sustaining Saudi Arabia’s momentum and global impact in personalized breast cancer therapy [66, 133].

### 4.7. Limitations

This study is limited by its specific time frame, which may not capture the full scope of anti–breast cancer drug discovery research in Saudi Arabia. In addition, reliance on publicly available academic data may exclude unpublished or industry‐led studies. Finally, these bibliometric criteria are more reflective of research productivity rather than direct clinical or societal impact, necessitating a caution interpretation of the findings.

## 5. Conclusion

Saudi Arabia has made significant strides in anti–breast cancer drug discovery driven by strategic investments, institutional support, and international collaborations. This bibliometric analysis highlights the notable growth of research activity, identifies key themes, and demonstrates leading Saudi institutions contributing to advancement in the field. The impact of Saudi Arabian research can be further enhanced by expanding research into emerging targets, integrating advanced technologies, and strengthening clinical application. Continued investment into research infrastructure, fostering collaborations, and promoting high‐quality studies are essential to advancing breast cancer research and contributing to global efforts aimed at developing effective cancer therapies.

## Ethics Statement

The authors have nothing to report.

## Conflicts of Interest

The authors declare no conflicts of interest.

## Author Contributions

Azizah M. Malebari conceptualization, data curation, formal analysis, investigation, methodology, project administration, supervision, writing–original draft, and writing–review & editing. Abdulrahman A. Alghelis data curation, formal analysis, validation, visualization, writing–original draft, and writing–—review and editing. Rashad S. Alzahrani data curation, software, writing–original draft, and writing–review and editing. Anfal S. Aljahdali resources, visualization, writing–original draft, and writing–review and editing.

## Funding

The authors declare that no financial support was received for the research, authorship, and publication of this article. Open Access publishing facilitated by the Deanship of Scientific Research (DSR) at King Abdulaziz University, as part of the Wiley — King Abdulaziz University agreement.

## Data Availability

The raw data supporting the conclusions of this article will be made available by the authors, without undue reservation.
